# Alteration of BDNF, SPARC, FGF-21, and GDF-15 circulating levels after 1 year of anti-obesity treatments and their association with 1-year weight loss

**DOI:** 10.1007/s12020-023-03435-2

**Published:** 2023-07-12

**Authors:** Kusuma Chaiyasoot, Nanta Khumkhana, Wanjan Deekum, Chartchai Chaichana, Voraboot Taweerutchana, Nicha Srisuworanan, Pornpoj Pramyothin

**Affiliations:** 1 Division of Nutrition, Department of Medicine, Mahidol University, Bangkok, Thailand; 2 Siriraj Center of Research Excellence for Diabetes and Obesity, Mahidol University, Bangkok, Thailand; 3 Division of Minimal Invasive Surgery, Department of Surgery, Faculty of Medicine Siriraj Hospital, Mahidol University, Bangkok, Thailand

## Abstract

**Purpose:**

Emerging evidence revealed that brain-derived neurotrophic factor (BDNF), secreted protein acidic and rich in cysteine (SPARC), fibroblast growth factor 21(FGF-21) and growth differentiation factor 15 (GDF-15) are involved in energy metabolism and body weight regulation. Our study aimed at examining their association with BMI, their alterations after anti-obesity treatments, and their association with 1-year weight loss.

**Methods:**

A prospective observational study of 171 participants with overweight and obesity and 46 lean controls was established. All participants received lifestyle educational intervention (LEI) with or without anti-obesity treatments (LEI + bariatric/metabolic surgery, *n* = 41; LEI + topiramate, *n* = 46; LEI + liraglutide, *n* = 31; LEI + orlistat, *n* = 12; and LEI alone, *n* = 41). Anthropometric and metabolic parameters, insulin sensitivity, C-reactive protein (CRP), fasting plasma levels of BDNF, SPARC, GDF-15, and FGF-21 were measured at baseline and 1 year.

**Results:**

Multiple linear regression showed that fasting levels of SPARC, FGF-21, and GDF-15 were significantly associated with baseline BMI after adjustment for age and sex. At 1 year, the average weight loss was 4.8% in the entire cohort with a significant improvement in glycemia, insulin sensitivity, and CRP. Multiple linear regression adjusted for age, sex, baseline BMI, type of treatment, and presence of T2DM revealed that the decrease in log_10_FGF-21 and log_10_GDF-15 at 1 year from baseline was significantly associated with a greater percentage of weight loss at 1 year.

**Conclusions:**

This study highlights the association of SPARC, FGF-21, and GDF-15 levels with BMI. Decreased circulating levels of GDF-15 and FGF-21 were associated with greater weight loss at 1 year, regardless of the types of anti-obesity modalities.

## Introduction

Body weight (BW) regulation is complex and requires interaction from various organs. Emerging evidence revealed that several cytokines [1–3] and gut hormones [4] played an important role in metabolism, energy balance, and BW regulation, and many of them were being developed as novel therapies for obesity. Brain-derived neurotrophic factor (BDNF), secreted protein acidic and rich in cysteine (SPARC), fibroblast growth factor 21 (FGF-21), growth differentiation factor 15 (GDF-15) are examples of such cytokines which have therapeutic potential in obesity and metabolic diseases.

BDNF is involved in neurotrophic activity, inflammation, metabolism, and cardiovascular diseases [5]. BDNF depletion in the hypothalamus [6], BDNF haploinsufficiency [7], and mutation of its receptors [8] have been reported to be related to increased dietary intake, weight gain, hence obesity. SPARC, also known as osteonectin or BM-40, is ubiquitously expressed in most tissues, especially subcutaneous fat. Recently, SPARC has also gained substantial interest due to its roles in obesity, insulin resistance, and metabolic syndrome [9].

FGF-21, secreted primarily from the liver, plays a crucial role in enhancing glucose uptake and lipolysis in adipose tissue, alleviating liver steatosis, and anti-inflammatory [10]. Furthermore, FGF-21 shifts food preference away from the sweet and high-calorie diet [11]. It also increases energy expenditure by activating brown and beige adipose tissue [2]. Therefore, FGF-21 is considered to have a novel therapeutic potential for obesity, T2DM, and NAFLD [2].

GDF-15, a member of the transforming growth factor β superfamily, is a stress-responsive cytokine [12]. GDF-15 is expressed in various tissues, mainly in the liver [1], and signals through a glial cell-derived neurotrophic factor receptor alpha-like (GFRAL) in the nucleus tractus solitarius and area prostrema [13], resulting in reduced food intake and BW, and improved glycemic control [14]. Therefore, the GDF-15/GFRAL axis is suggested as an essential part of energy homeostasis and BW regulation and is currently a novel therapeutic target for obesity.

Few studies examine these cytokines in people who are obese or overweight. Furthermore, research that describes alterations in circulating levels of these cytokines in response to anti-obesity treatments including pharmacotherapy or bariatric/metabolic surgery is scarce. This information can shed light on the role of these cytokines in the pathogenesis of obesity and could lead to the development of novel obesity treatments.

Thus, the present study aimed to study the association of circulating levels of BDNF, SPARC, FGF-21, and GDF-15 with BMI, the alterations of these four cytokines after 1 year of anti-obesity treatments (lifestyle modification plus pharmacotherapy or bariatric/metabolic surgery), and their association with weight loss at 1 year after obesity therapy.

## Materials and methods

### Study participants

A prospective cohort of 171 adults with overweight (BMI 23–24.9 kg/m^2^), obesity (BMI ≥ 25 kg/m^2^) and 46 lean controls (BMI 18.5–22.9 kg/m^2^) was established. The BMI criterion used in this study is ethnically specific for Asians [15]. All participants were ≥18 years old and were not pregnant or lactating. The controls reported no history of obesity, diabetes mellitus, high blood pressure, or dyslipidemia, and were in good health. Written informed consent was given by all participants. The study was approved by the respective Institutional Ethics Committee for Clinical Research of Siriraj Hospital, Mahidol University, Thailand, and was conducted at Siriraj Hospital, Faculty of Medicine Siriraj Hospital from 2017 to 2019. Study data was collected and managed using REDCap electronic data capture tools [16].

All participants with obesity received lifestyle educational intervention (LEI) with or without pharmacotherapy (for participants with BMI ≥27 kg/m^2^ with obesity-associated comorbidities or BMI ≥30 kg/m^2^) including topiramate, liraglutide, and orlistat, or bariatric/metabolic surgery (for participants with BMI ≥35 kg/m^2^ with obesity-associated comorbidities or BMI ≥40 kg/m^2^). The option of anti-obesity treatments was chosen based on specific contraindications for each individual. The decision was made by the participants and an attending physician after a detailed discussion. LEI and pharmacotherapy were delivered to participants throughout the 1-year period of the study.

At baseline, participants were divided into four groups according to their BMI: lean control (BMI 18.5–22.9 kg/m^2^), group 1 (BMI 25–29.9 kg/m^2^), group 2 (BMI 30–39.9 kg/m^2^) and group 3 (BMI ≥40 kg/m^2^).

### Anti-obesity treatments

#### Lifestyle education intervention (LEI)

Individual sessions of lifestyle education were provided by a registered dietitian or an obesity physician at baseline and at each follow-up visit. The session focused on reduced energy intake, targeting a deficit of 2093–4186 kJ (500–1000 kcal) per day; and regular exercise, aiming for at least 150 min of moderate intensity exercise per week and/or 10,000 steps per day.

#### LEI + topiramate

The dose of topiramate was titrated in the first 4 weeks of the intervention as follows: the first week, 25 mg before bedtime once a day; the second week, 25 mg twice a day after breakfast and before bedtime; the third week, 50 mg twice a day after breakfast and before bedtime; the fourth week, 50 mg after breakfast and 100 mg before bedtime. However, if the 150 mg dose could not be tolerated, the dose would decreased to a maximum tolerable dose.

#### LEI + liraglutide

Liraglutide was administered by subcutaneous injection. The starting dose was 0.6 mg per day and then the dose increased by 0.6 mg each week until the dose of 3.0 mg was reached. However, if the 3.0-mg dose could not be tolerated, the dose would be deescalated to a maximal tolerable dose.

#### LEI + orlistat

Orlistat was prescribed at a dose of 120 mg 2–3 times a day with meals.

#### LEI + bariatric/metabolic surgery

Laparoscopic roux-en-Y gastric bypass (LRYGB) included the construction of a biliopancreatic limb with a 100- to 120-cm alimentary limb and a 30-ml gastric pouch. In laparoscopic sleeve gastrectomy (LSG), approximately 80% of the stomach is removed, producing a narrow tubular stomach that leads to rapid gastric emptying and nutrients passing rapidly into the duodenum and proximal part of the small intestine.

### Outcomes measurement

Demographic data including age, sex, height, and comorbidities were collected from the hospital’s electronic database. The BW was recorded at each visit. Waist circumference (WC) and hip circumference (HC) were measured at baseline and 1 year. Total cholesterol (TC), triglyceride (TG), high-density lipoprotein cholesterol (HDL-c), fasting plasma glucose (FPG), hemoglobin A1c (HbA_1c_), fasting insulin, high-sensitivity C-reactive protein (CRP), and fasting plasma levels of BDNF, SPARC, FGF-21, and GDF-15 were measured at baseline and 1 year.

### Anthropometric measurement

The BW was measured using a calibrated weighing scale (TANITA^®^ BC-587, Central trading Co., Ltd, Thailand). Participants were weighed while wearing indoor clothing without shoes and heavy accessories. BMI was calculated by BW (kg)/(height [m])^2^. WC and HC were measured using non-stretchable tape. WC was measured midway between the iliac crests and the lowest ribs. HC was measured at the widest protrusion of the buttocks. The W/H ratio was calculated by WC/HC. The percentage of weight loss (PWL) was determined by ([baseline BW – BW at 1 year]/ baseline BW) x 100.

### Biochemical analysis

A venous blood sample was taken after a fast for 12 h overnight. TC, HDL-c, LDL-c, TG, glucose, and insulin were determined by a biochemical auto-analyzer (Cobas® 8000 modular analyzer series, Roche Diagnostics, Indianapolis, USA). HbA_1c_ was analyzed with Cobas Integra® 800 analyzer, Roche Diagnostics, Indianapolis, USA. LDL-c levels were calculated using the Friedewald formula. Homeostatic model assessment insulin resistance (HOMA-IR) was calculated using the following formula: (FPG × fasting insulin)/22.5 in molar units.

For cytokine analysis, EDTA plasma was separated by centrifugation at 3500 rpm for 10 min. Plasma was then collected and stored at −80 °C, awaiting analysis for BDNF, SPARC, FGF-21, and GDF-15. Plasma levels of BDNF, SPARC, FGF-21, and GDF-15 were quantified using a bead-based multiplex assay kit (MILLIPLEX MAP Human Myokine Magnetic Bead Panel, Merck, Germany) according to the manufacturer’s instructions (respectively, intra-assay variability: <10%, <10%, <5%, <10%, and inter-assay variability: <15%, <20%, <15%, <10%).

### Statistical analysis

Statistical analyzes were performed using SPSS version 18.0 software. The normal distribution of the variables was explored using the Kolmogorov–Smirnov and Shapiro–Wilk tests. Skewed distributions were logarithmically transformed for comparison. Unpaired and paired sample *t*-tests were used for comparisons in two unrelated samples and related sample groups, respectively. Analysis of variance (ANOVA) was used to study the difference between groups. The results of the quantitative variables were expressed as mean ± standard deviation for the normally distributed variables, and as median and interquartile ranges for the nonnormally distributed variables. The association of cytokines with BMI was tested using a multiple linear regression analysis adjusted for age and sex. The relationship between plasma cytokines and PWL was analyzed using a multiple linear regression model adjusted for age, sex, baseline BMI, type of anti-obesity treatments, and presence of T2DM. A *p*-value < 0.05 was considered statistically significant in all analyzes.

## Results

### Baseline characteristics

Table 1 presents clinical characteristics and fasting plasma levels of cytokines according to the BMI groups at baseline. Overall, the average age was 39 years. The majority of the participants were female (62.7%), and 26.3% of the participants had T2DM. There was an increasing number of participants with T2DM, HT, dyslipidemia (DLP), and obstructive sleep apnea (OSA) according to the increasing BMI. CRP levels also increased significantly along with the increase in BMI groups (*P* < 0.001).

**Table 1:** Clinical characteristics and fasting plasma levels of cytokines according to BMI groups at baseline

Parameters	Lean control (*n* = 46) BMI 18.5–22.9 kg/m^2^	Group 1 (*n* = 14) BMI 25–29.9 kg/m^2^	Group 2 (*n* = 67) BMI 30–39.9 kg/m^2^	Group 3 (*n* = 90) BMI ≥ 40 kg/m^2^	*P*-value
Age, years	35.1 ± 8.7	42.3 ± 16.4	42.7 ± 13.4	36.6 ± 12.8	0.005
Female, *n* (%)	23 (50.0)	14 (100)	42 (62.7)	57 (63.3)	0.02
Type 2 diabetes mellitus, *n* (%)	0	1 (7.1)	17 (25.4)	27 (30.0)	0.19
Hypertension, *n* (%)	0	3 (21.4)	32 (47.8)	48 (53.3)	0.08
Dislipidemia, *n* (%)	0	2 (14.3)	19 (28.4)	26 (28.9)	0.51
Obstructive sleep apnea, *n* (%)	0	0	14 (25.4)	44 (48.9)	<0.001
Inflammatory marker					
CRP, mg/l	0.59 (0.30,1.14)	1.07 (0.86, 2.29)	3.63 (1.75, 5.72)	8.03 (4.75,13.19)	<0.001
Cytokine concentrations					
BDNF, pg/ml	475 (234, 1095)	759 (412, 1344)	670 (369, 1414)	646 (380, 1553)	0.12
SPARC, ng/ml	24.8 (17.2, 30.4)	42.1 (35.7, 51.1)	55.1 (41.2, 82.5)	58.6 (43.4, 75.3)	<0.001
FGF-21, pg/ml	4.8 (2.21 10.7)	10.3 (4.7, 20.6)	18.7 (9.2, 33.8)	24.6 (12.2, 49.8)	<0.001
GDF-15, pg/ml	420 (274, 532)	441 (323, 724)	777 (578, 1309)	968 (616, 1650)	<0.001

Data are expressed as mean ± SD or median (interquartile range) according to data distribution

**Fig. 1:**
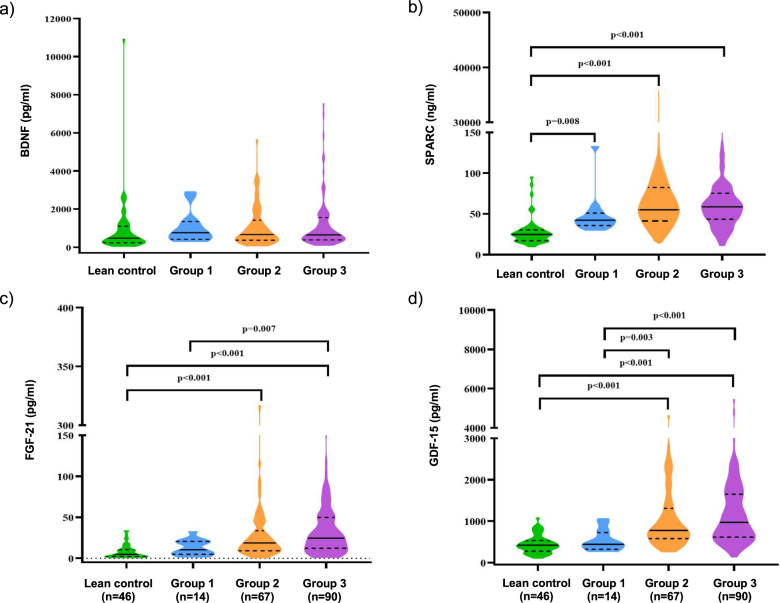
Comparison of plasma cytokine concentrations in different BMI groups; lean control (BMI = 18.5–22.9 kg/m^2^), group 1 (BMI = 25.0–29.9 kg/m^2^), group 2 (BMI = 30.0–39.9 kg/m^2^) and group 3 (BMI ≥ 40 kg/m^2^). **a** BDNF, **b** SPARC, **c** FGF-21 and **d** GDF-15

The fasting plasma levels of BDNF were not significantly different between groups (*P* = 0.12, Table 1 and Fig. 1a). The fasting plasma levels of SPARC in BMI group 1, group 2, and group 3 were significantly higher than lean control (*P* < 0.01, *P* < 0.001, and *P* < 0.001, respectively, Table 1, Fig. 1b). The fasting plasma levels of FGF-21 in BMI group 2 and group 3 were significantly higher than lean control (*p* < 0.001 for both), and levels in BMI group 3 were significantly higher than BMI group 1 (*p* < 0.01) (Table 1 and Fig. 1c). Circulating GDF-15 levels in BMI group 2 and BMI group 3 were significantly higher than lean control (*P* < 0.001 for both), and levels in BMI group 2 and BMI group 3 were significantly higher than BMI group 1 (*P* < 0.01 and *P* < 0.001) (Table 1 and Fig. 1d).

**Fig. 2:**
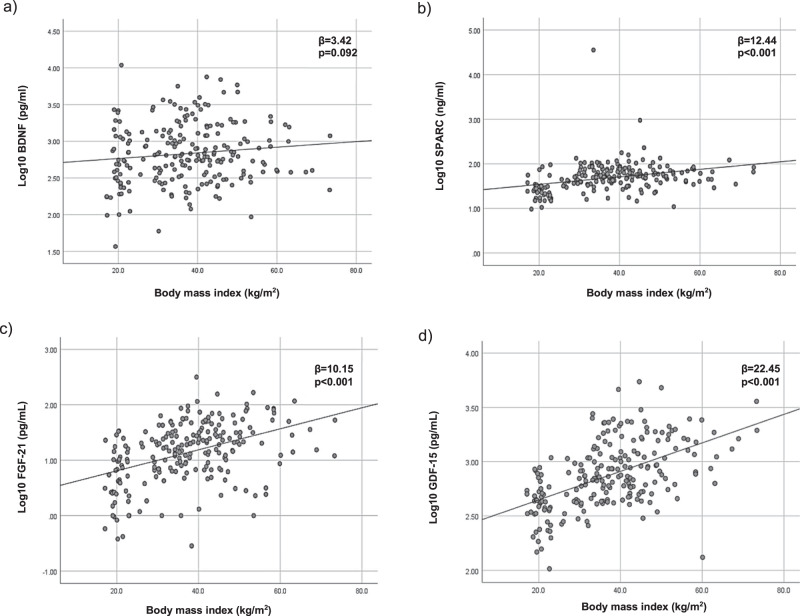
Association of cytokines with BMI by a multiple linear regression analysis adjusted for age and sex. **a** Log10 BDNF, **b** Log10 SPARC, **c** Log10 FGF-21 and **d** Log10 GDF-15

Multiple linear regression also showed that log10SPARC, log10FGF-21, and log10GDF-15 were significantly associated with BMI after adjusting for age and sex (*β* = 12.4, *P* < 0.001 for log10SPARC; *β* = 10.2, *P* < 0.001 for log10FGF-21; *β* = 22.5, *P* < 0.001 for log10GDF-15) (Fig. 2). There was no relationship between log10 BDNF levels and BMI at baseline.

### Changes in parameters at 1 year

#### Anthropometric parameters

In the entire cohort, there was a significant decrease in BW, BMI, and WC at 1 year from baseline (*P* < 0.001 for all, Table 2). BW and BMI were significantly reduced in LEI + topiramate (*P* < 0.001 for both), BW and WC decreased significantly in LEI + liraglutide (*P* < 0.001 and *P* < 005, respectively), and the BW, BMI, WC and W/H ratio were significantly reduced in LEI + bariatric/metabolic surgery (*P* < 0.001 for all) (Table 2). The average PWL at 1 year was 4.8% with the highest PWL in LEI + bariatric/metabolic surgery (21.6%), 5.7% in LEI + topiramate, 3% in LEI + liraglutide and 0.8% in LEI alone. In contrast, LEI + orlistat experienced a weight gain of 2% (Table 2).

**Table 2:** Changes in anthropometric parameters, metabolic indices, inflammatory marker and cytokine concentrations in subjects with overweight and obesity at 1 year from baseline, divided by type of anti-obesity treatments

Parameters	Total (*n* = 171)		*P*	LEI (*n* = 41)		*P*	LEI + topiramate (*n* = 46)		*P*	LEI + liraglutide (*n* = 31)		*P*	LEI + orlistat (*n* = 12)		P	LEI + bariatric/metabolic surgery (*n* = 41)		*P*
	Baseline	1 year		Baseline	1 year		Baseline	1 year		Baseline	1 year		Baseline	1 year		Baseline	1 year	
Anthropometric parameters																		
BW, kg	112 ± 31	101 ± 27	<0.001	106 ± 27	105 ± 27	0.10	109 ± 32	101 ± 32	<0.001	94.6 ± 18.5	90.8 ± 18.1	<0.001	114 ± 32	116 ± 34	0.61	134 ± 31	102 ± 26	<0.001
BMI, kg/m^2^	41.7 ± 9.8	37.7 ± 8.4	<0.001	39.1 ± 8.3	38.6 ± 8.5	0.10	40.3 ± 9.5	37.2 ± 9.5	<0.001	36.5 ± 5.2	35.7 ± 6.7	0.28	40.6 ± 9.6	41.2 ± 10.8	0.58	50.2 ± 9.3	38.0 ± 7.4	<0.001
WC, cm	119 ± 21	114 ± 18	<0.001	114 ± 16	113 ± 16	0.47	119 ± 25	116 ± 23	0.38	114 ± 15	110 ± 13	0.03	122 ± 25	122 ± 24	1.00	132 ± 21	113 ± 16	<0.001
W/H ratio	0.93 ± 0.09	0.92 ± 0.07	0.29	0.91 ± 0.08	0.91 ± 0.08	0.85	0.93 ± 0.11	0.93 ± 0.08	0.96	0.94 ± 0.06	0.92 ± 0.05	0.11	0.92 ± 0.08	0.94 ± 0.07	0.45	0.93 ± 0.08	0.91 ± 0.07	<0.001
PWL, %	–	4.76 (0.8, 16.2)	–	–	0.78 (−1.4, 3.1)	–	–	5.65 (1.7, 10.8)	–	–	3.01 (0.5, 8.0)	–	–	−1.97 (−5.5, 5.3)	–	–	21.6 (18.6, 29.3)	–
Glycemic and insulin sensitivity indices																		
FPG, mmol/L	6.01 ± 1.69	5.52 ± 1.51	<0.001	5.50 ± 1.07	5.35 ± 1.30	0.38	5.76 ± 1.15	5.60 ± 1.72	0.53	6.25 ± 1.58	6.14 ± 1.44	0.66	5.87 ± 0.96	5.00 ± 1.68	0.21	6.66 ± 2.54	5.25 ± 1.32	<0.001
HbA1c, %	6.09 ± 1.05	5.75 ± 0.76	<0.001	5.67 ± 0.57	5.74 ± 0.81	0.37	6.0 ± 0.8	5.8 ± 0.8	0.30	6.19 ± 0.91	6.03 ± 0.63	0.16	5.89 ± 0.48	5.78 ± 0.59	0.37	6.63 ± 1.58	5.44 ± 0.69	<0.001
Insulin, pmol/L	147 (98, 219)	101 (66, 185)	<0.001	129 (89, 190)	105 (68, 175)	0.23	139 (96, 218)	133 (75, 245)	0.24	158 (94, 205)	149 (81, 220)	0.72	152 ± 74	156 ± 103	0.90	228 ± 153	81.1 ± 48.1	<0.001
HOMA-IR	2.70 (1.85, 4.15)	2.00 (1.20, 3.40)	<0.001	2.30 (1.70, 3.40)	2.00 (1.30, 3.20)	0.25	2.50 (1.80, 4.05)	2.55 (1.45, 4.37)	0.33	2.90 (1.80, 3.70)	2.95 (1.55, 4.12)	0.65	2.70 (1.93, 3.80)	1.80 (1.20, 3.70)	0.79	2.70 (1.85, 4.15)	2.00 (1.20, 3.40)	<0.001
Lipid profile																		
TC, mg/dl	180 ± 36	179 ± 38	0.73	183 ± 32	180 ± 32	0.36	175 ± 34	184 ± 47	0.10	178 ± 39	179 ± 38	0.86	182 ± 31.3	189 ± 29.4	0.40	183 ± 43	170 ± 35	0.048
TG, mg/dl	122 (95, 171)	112(810, 156)	0.03	121 ± 45	123 ± 62	0.83	140 (104, 204)	132 (90, 167)	0.50	138 ± 51	138 ± 52	0.91	128 ± 60	122 ± 39	0.63	135 ± 66	70.0 ± 371	0.001
HDL-c, mg/dl	46.6 ± 12.8	50.2 ± 34.5	<0.001	47.9 ± 11.7	49.4 ± 13	0.11	43.0 ± 12.2	48.5 ± 15.1	0.00	48 ± 13	51 ± 16	0.00	52.2 ± 16.3	49.9 ± 13.2	0.19	47.3 ± 12.9	51.9 ± 11.2	0.002
LDL-c, mg/dl	106 ± 35	102 ± 33	0.07	112 ± 31	106 ± 30	0.10	101 ± 29	101 ± 29	0.98	104 ± 36	100 ± 39	0.34	104 ± 29	114 ± 33	0.23	107.9 ± 42.8	96.6 ± 36.2	0.073
Inflammatory marker																		
CRP, mg/l	5.03 (2.51, 9.58)	3.32 (1.62, 5.52)	<0.001	5.03 (2.51, 9.58)	3.32 (1.62, 6.62)	0.65	4.15 (2.16, 7.40)	3.47 (1.63, 6.71)	0.10	4.68 (1.38, 11.11)	2.66 (1.19, 7.55)	0.72	3.36 (1.79, 7.09)	6.06 (2.63, 9.37)	0.25	8.58 (4.93, 12.44)	3.05 (1.13, 5.12)	<0.001
Cytokine concentrations																		
BDNF, pg/ml	663 (381, 1376)	680 (428, 1555)	0.75	611 (280, 1190)	535 (331, 1136)	0.74	935 (539, 1851)	918 (544, 2202)	0.39	548 (319, 984)	551 (384, 1636)	0.65	754 (442, 1330)	613 (482, 1403)	0.48	706 (351, 1425)	734 (499, 1164)	0.96
SPARC, ng/ml	55.0 (41.4, 76.5)	39.9 (28.9, 56.2)	<0.001	37.6 (27.0, 50.8)	33.9 (22.5, 46.0)	0.07	55.1 (43.3, 83.2)	46.2 (31.3, 64.8)	0.002	80.8 (66.2, 104.2)	41.0 (31.1, 64.1)	<0.001	45.8 (39.8, 53.5)	47.5 (39, 140)	0.70	58.6 (48.7, 71.0)	38.6 (27.1, 53.4)	<0.001
FGF-21, pg/ml	20.7 (9.3, 42.8)	18.8 (9.9, 33.8)	0.70	15.4 (7.1, 26.5)	18.7 (9.8, 31.8)	0.08	24.4 (11.1, 51.3)	21.3 (10.0, 55.3)	0.78	12.8 (6.7, 29.2)	19.4 (7.1, 36.5)	0.12	18.6 (12.9, 45.0)	21.5 (12.3, 40.6)	0.81	31.4 (14.0, 54.0)	14.7 (9.9, 26.4)	0.005
GDF-15, pg/ml	853 (572, 1515)	770 (506, 1201)	0.052	676 (453, 1061)	817 (545, 1184)	0.012	1041 (650, 2075)	974 (506, 1472)	0.021	701 (451, 868)	706 (540, 1148)	0.38	673 (496, 1448)	706 (498, 1040)	0.70	1165 (674, 1864)	606 (444, 1381)	0.003

#### Metabolic and insulin sensitivity indices

In all participants, there was a significant decrease in TG levels (*P* < 0.05) and a significant increase in HDL-c levels (*P* < 0.001) (Table 2). In the LEI + bariatric/metabolic surgery group, participants had significantly reduced TG and total cholesterol levels (*P* = 0.001 and *P* < 0.05), and increased HDL-c levels (*P* < 0.01) (Table 2). There was a significant increase in HDL-c levels in LEI + topiramate and LEI + liraglutide (*P* < 0.01 for both) (Table 2).

FPG, HbA_1c_, fasting insulin and HOMA-IR levels decreased significantly at 1 year, compared to baseline only in the LEI + bariatric/metabolic surgery group (*P* < 0.001 for all) (Table 2).

#### Inflammatory marker

Circulating CRP levels were significantly reduced at 1 year in LEI + bariatric/metabolic surgery group (*P* < 0.001) (Table 2).

#### Cytokines

**Fig. 3:**
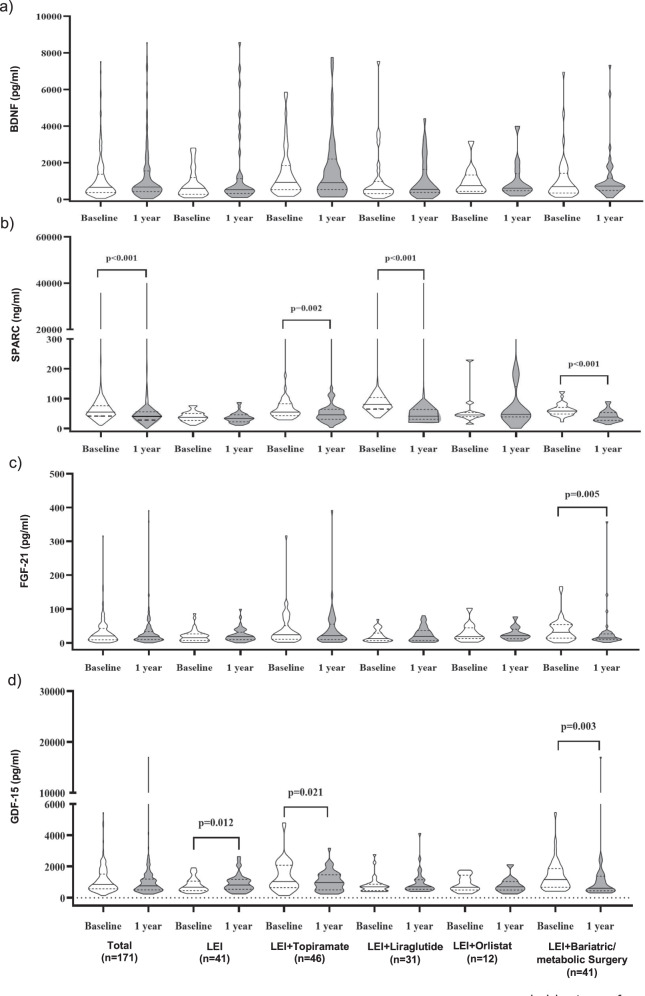
Changes in plasma cytokine concentrations at 1 year from baseline divided by type of anti-obesity treatments. **a** BDNF, **b** SPARC, **c** FGF-21 and **d** GDF-15; LEI lifestyle education intervention

At 1 year, BDNF levels were not significantly different from baseline in all groups (Table 2 and Fig. 3a). Circulating SPARC levels were significantly lower than baseline in the entire cohort (*P* < 0.001), LEI + topiramate (*P* < 0.01), LEI + liraglutide (*P* < 0.001), and LEI + bariatric/metabolic surgery (*P* < 0.001) (Table 2 and Fig. 3b). The levels of FGF-21 significantly reduced in LEI + bariatric/metabolic surgery at 1 year (*P* < 0.01) (Table 2 and Fig. 3c). Circulating levels of GDF-15 decreased significantly in LEI + topiramate (*P* < 0.05) and LEI + bariatric/metabolic surgery (*P* < 0.01) (Table 2 and Fig. 3d).

### Association study of cytokine levels with 1-year PWL

Multiple linear regression adjusted for age, sex, baseline BMI, type of anti-obesity treatments, and presence of T2DM was performed to examine an association between cytokine levels and 1-year PWL. It revealed that the decrease in log_10_FGF-21 and log_10_GDF-15 at 1 year from baseline (∆log_10_FGF-21 and ∆log_10_GDF-15) were significantly associated with greater 1-year PWL (*β* = −4.4, *P* < 0.001 and *β* = −4.5, *P* < 0.05, respectively, Table 3).

**Table 3:** Association study of plasma cytokines levels with 1-year percentage weight loss, adjusted for age, sex, baseline BMI, type of anti-obesity treatments and the presence of T2DM

Parameters	*β*	95% confidence interval of *β*		*R*^2^	*P*-value
		Lower bound	Upper bound		
At baseline					
Log10 BDNF, pg/ml	−0.63	−3.43	2.17	0.63	0.66
Log10 SPARC, ng/ml	−0.62	−4.60	3.36	0.63	0.76
Log10 FGF-21, pg/ml	1.04	−1.33	3.40	0.63	0.39
Log10 GDF-15, pg/ml	−1.71	−6.16	2.75	0.63	0.45
Changes at 1 year from baseline					
ΔLog10 BDNF, pg/ml	0.07	−2.19	2.33	0.63	0.95
ΔLog10 SPARC, ng/ml	0.46	−2.31	3.24	0.63	0.74
ΔLog10 FGF-21, pg/ml	−4.39	−6.62	−2.16	0.66	<0.001
ΔLog10 GDF-15, pg/ml	−4.49	−8.46	−0.52	0.64	0.027

## Discussion

The present study demonstrated that circulating levels of SPARC, FGF-21, and GDF-15 but BDNF were positively correlated with BMI. At 1 year after anti-obesity treatments, there was a significant reduction in BW (average PWL = 5%), BMI and WC, and a significant improvement in glycemia, insulin sensitivity indices, and CRP levels. Circulating SPARC levels were significantly lower than baseline in LEI + topiramate, LEI + liraglutide, and LEI+bariatric/metabolic surgery. Circulating FGF-21 levels were significantly reduced in LEI + bariatric/metabolic surgery. Circulating GDF-15 levels decreased significantly in LEI + topiramate and LEI + bariatric/metabolic surgery. There was no significant change in BDNF circulating levels at 1 year from baseline. Furthermore, we discovered that decreasing levels of circulating FGF-21 and GDF-15 were associated with greater weight loss at one year regardless of the type of anti-obesity therapies.

Despite the advantages of BDNF in BW, a systematic review and meta-analysis revealed that there was no difference in circulating levels of BDNF between people with obesity and lean controls [5]. This is in agreement with our findings that there was no significant association between plasma levels of BDNF and BMI. Furthermore, BDNF levels at 1 year after anti-obesity treatments were comparable to baseline levels. The reasons for these results could be: first, circulating levels of BDNF may not truly represent BDNF levels in the hypothalamus, which is the key organ regulating BW; second, the main source of circulating BDNF is still poorly understood (different sources could have different functions) [17]; and third, it has been reported that there was a lack of standard protocol for collecting and processing plasma and/or serum BDNF [5].

The concept of adipose expandability [18] describes that adipose expansion is vital for coping with surplus energy intake. When expandability is restricted, excess TG enters the circulation, contributing to hyperlipidemia and ectopic fat accumulation. Consequently, this results in insulin resistance and metabolic syndrome. SPARC is claimed to be responsible for adipose tissue fibrosis, thus restricting adipose tissue expandability and adipogenesis [19]. However, recent evidence showed that SPARC had advantages on energy metabolism. SPARC increased thermogenesis through brown adipose tissue [20] and increased energy expenditure in skeletal muscle [21].

In the present study, we found that SPARC levels were significantly associated with BMI. This could be explained by: first, higher levels of inflammation, insulin resistance, leptin, fat mass at a higher BMI associated with higher levels of SPARC, as reported by several previous studies [9, 20, 22, 23]; second, a body’s attempt to limit expansion of adipose tissue at a higher BMI; and third, a compensation to increase energy expenditure through brown and beige adipocytes and skeletal muscles.

After 1 year of anti-obesity treatments, SPARC levels decreased significantly throughout the cohort, LEI + topiramate, LEI + liraglutide, and LEI + bariatric/metabolic surgery, where they were groups that showed significant weight loss. This corresponds to previous studies revealing that a gene encoding SPARC could be down-regulated by energy restriction in mice [24], and a very low-calorie diet reduced SPARC expression by 33% in humans [9]. Furthermore, two studies that examined SPARC concentrations after bariatric/metabolic surgery showed that there was a significant decrease in SPARC levels [9, 25]. The significant reduction in SPARC levels after anti-obesity treatments in our study could be due to improved metabolic abnormalities and inflammation associated with obesity. Therefore, elevated levels of SPARC to counteract the obesity state and its complications may no longer be needed. The direct effects of topiramate and liraglutide on SPARC concentrations are not well understood.

Interestingly, Lee et al. reported that changes in serum SPARC levels after bariatric/metabolic surgery were significantly correlated with changes in HOMA-IR, not BMI [25]. This is in line with our findings that changes in circulating SPARC did not show an association with 1-year PWL in a multiple linear regression adjusting for age, sex, baseline BMI and the presence of T2DM.

At baseline, we found that FGF-21 circulating levels were significantly associated with BMI. This confirms that FGF-21 secretion is influenced by physiological or environmental stress in people with obesity, as reported in previous studies [26]. Interestingly, an FGF-21 resistant state in obesity could be another reason. A previous study in diet-induced obesity mice demonstrated that there was decreased expression of the FGF-21 receptor in white adipose tissue and after FGF-21 administration, the reduction in plasma glucose was attenuated, compared to lean ones [27]. A study in people living with obesity and T2DM demonstrated that the expression of genes comprising the FGF-21 signaling pathway was also lower in visceral fat than in subcutaneous fat. They concluded that human FGF-21 resistance in T2DM and obesity could result from increases in FGF-21-resistant ectopic fat accumulation [28].

Our results revealed that decreasing levels of FGF-21 at 1 year from baseline were significantly associated with higher PWL. This was mainly driven by the group ‘LEI + bibariatric/metabolic surgery’, as the reduction in 1-year levels of FGF-21 was the most striking in this group. Changes in FGF-21 levels in response to bariatric/metabolic surgery are reportedly varied and inconclusive. A meta-analysis by Hosseinzadeh et al. indicated that the alteration of fasting FGF-21 levels was dominantly affected by the duration of follow-up [10]. Fasting levels of FGF-21 increased significantly after RYGB, particularly in the early post-op; however, the levels decreased considerably at ≥ 1 year follow-up duration. This supports our findings that fasting FGF-21 levels decreased significantly at 1 year after bariatric/metabolic surgery.

The proposed mechanisms that explain the reduction in 1-year levels of circulating FGF-21 include: first, resolved resistance status to FGF-21 after a significant weight reduction; second, improved metabolic stress as FGF-21 is a stress-induced cytokine; and third, significantly reduced food intake, as FGF-21 is strictly nutritionally controlled [10, 29]. Evidence in rats revealed that there was an improvement in FGF-21 sensitivity, as well as restoration of the FGF-21 signaling pathway after SG and duodenal-jejunal bypass at 1 year [30]. Furthermore, an up-regulation of FGF-21 receptors in adipose tissue has been reported in humans after RYGB [31].

GDF-15 concentrations have been reported to positively correlate with age, BMI, W/H ratio, adiposity, glucose, degrees of insulin resistance, and CRP [13, 32]. This is in agreement with our results showing that GDF-15 levels were significantly associated with BMI at baseline.

Studies in mice revealed that GDF-15 causes weight loss and taste aversion away from high-calorie food [12, 33–36]. Li et al. discovered that GDF-15 could prevent endothelial cell injury and cell apoptosis from high plasma glucose [37]. In addition, obesity and T2DM are known to manifest themselves as a systemic inflammatory state. Therefore, in addition to being simply a cell/tissue stress-induced cytokine, it is plausible that the role of higher levels of GDF-15 in obesity and diabetes is to prevent progressive weight gain and to play a role in reducing inflammation [12, 13].

In the present study, at 1 year, the reduction in GDF-15 levels was statistically significant in LEI + topiramate and LEI+baritric/metabolic surgery where they were the two main weight reduction strategies in this cohort (PWL = 21.6% and 5.7%, respectively). The magnitude of the reduction in GDF-15 was highest in LEI + bariatric/metabolic surgery, where the weight loss was also the greatest. This could indicate that at least 5% weight loss is required for the reduction in GDF-15 concentrations.

Furthermore, a multiple linear regression analysis suggests that the reduction in GDF-15 at 1 year from baseline was associated with a greater PWL after anti-obesity treatments. This could be explained by weight loss that leads to a reduced inflammatory burden and a substantial decrease in the need for GDF-15 to prevent progressive weight gain, thus a decrease in circulating GDF-15 levels.

Our findings ask whether or not GDF-15 is a key mediator of weight loss after anti-obesity treatments, particularly bariatric/metabolic surgery. Frikke-Schmidt et al. reported that deletion of *Gdf15* did not affect weight loss and feeding behavior after SG in mice [36]. Adolph et al. also showed that weight loss with laparoscopic adjustable gastric banding was related to decreased expression of *Gdf15* in the liver [38].

On the contrary, several studies revealed that GDF-15 levels increased after SG [39, 40] and RYGB [32, 41] and were positively correlated with weight loss, suggesting that GDF-15 could be a key mechanism for weight loss benefit after bariatric/metabolic surgery. However, the levels of GDF-15 at baseline in these studies (215 – 487 pg/ml) were lower than in our study (1165 pg/ml). Previous studies have shown that there was variability in GDF-15 response of anti-obesity treatments. After a week of daily 60-min aerobic exercise training, GDF-15 levels increased in 67% (6/9) of participants and reduced in 33% (3/9) of participants [42]. Furthermore, a 3-week lifestyle intervention caused an increase in GDF-15 in 77% and a decrease in 23% of the total participants [43]. This emphasizes the differences between individuals in the physiology and secretion profiles of GDF-15 in response to the obesity intervention. The effect of topiramate on GDF-15 concentrations is currently unknown.

The strengths of the present study include: first, it is a large sample size cohort of people with overweight and obesity, reporting circulating levels of BDNF, SPARC, FGF-21 and GDF-15 before and after obesity therapy; second, we demonstrated changes in circulating levels of the four cytokines in response to several anti-obesity treatments, including LEI with or without pharmacotherapy or bariatric/metabolic surgery; and third, the study participants were Asian in origin where this kind of study is still lacking.

Some limitations are worth noting. First, a small sample size in the LEI + orlistat group led to inconclusive results in this group; second, the cytokine levels were not measured at each follow-up visit between baseline and 1 year, making the study of the temporal relationship of changes impossible; and third, pharmacotherapy used in the present study reflects local practice context in Thailand; therefore, other pharmacotherapy options were not evaluated.

In conclusion, circulating levels of SPARC, FGF-21, and GDF-15 were positively correlated with BMI, and after weight reduction therapy, these levels were reduced according to weight loss. Furthermore, decreasing levels of FGF-21 and GDF-15 were associated with greater weight loss at 1 year. This could indicate that in the obesity state, cytokines are released in response to stress and inflammation and could function to prevent further weight gain and metabolic derangements. In the weight-reduced state, the improvement in inflammation, stress, and metabolic abnormalities probably results in reduced levels of the cytokines. Further research should focus on how these cytokines function in a weight-reduced state and how they respond to anti-obesity treatments.
